# Mixed Cardiogenic-Vasodilatory Shock: Current Insights and Future Directions

**DOI:** 10.1016/j.jacadv.2024.101432

**Published:** 2024-12-05

**Authors:** Jacob C. Jentzer, David D. Berg, Meshe D. Chonde, Garima Dahiya, Andrea Elliott, Penelope Rampersad, Shashank S. Sinha, Alexander G. Truesdell, Seife Yohannes, Saraschandra Vallabhajosyula

**Affiliations:** a Department of Cardiovascular Medicine, Mayo Clinic, Rochester, Minnesota, USA; b Cardiovascular Division, Department of Medicine, Brigham and Women’s Hospital, Harvard Medical School, Boston, Massachusetts, USA; c Department of Cardiology, Smidt Heart Institute, Cedars Sinai, Los Angeles, California, USA; d Division of Pulmonary and Critical Care Medicine, Duke University, Durham, North Carolina, USA; e Department of Cardiology, University of Minnesota, Minneapolis, Minnesota, USA; f Department of Cardiology, NYU Langone Health, New York, New York, USA; g Inova Schar Heart and Vascular, Inova Fairfax Medical Campus, Falls Church, Virginia, USA; h Department of Critical Care Medicine, Medstar Washington Hospital Center, Washington DC, USA; i Division of Cardiology, Department of Medicine, Warren Alpert Medical School of Brown University, and Lifespan Cardiovascular Institute, Providence, Rhode Island, USA

**Keywords:** cardiac arrest, cardiac intensive care unit, cardiogenic shock, myocardial infarction, sepsis, shock

## Abstract

This state-of-the-art review describes the potential etiologies, pathophysiology, and management of mixed shock in the context of a proposed novel classification system. Cardiogenic-vasodilatory shock occurs when cardiogenic shock is complicated by inappropriate vasodilation, impairing compensatory mechanisms, and contributing to worsening shock. Vasodilatory-cardiogenic shock occurs when vasodilatory shock is complicated by myocardial dysfunction, resulting in low cardiac output. Primary mixed shock occurs when a systemic insult triggers both myocardial dysfunction and vasoplegia. Regardless of the etiology of mixed shock, the hemodynamic profile can be similar, and outcomes tend to be poor. Identification and treatment of both the initial and complicating disease processes is essential along with invasive hemodynamic monitoring given the evolving nature of mixed shock states. Hemodynamic support typically involves a combination of inotropes and vasopressors, with few data available to guide the use of mechanical circulatory support. Consensus definitions and novel treatment strategies are needed for this dangerous condition.

## Highlights

• Mixed cardiogenic-vasodilatory shock is common in the contemporary cardiac intensive care unit.
• Patients can develop mixed shock from a primary cardiac, vascular, or systemic cardiovascular insult.
• Support of cardiac output and systemic vascular resistance is needed to treat mixed shock.
• Further research is needed to identify disease-modifying therapies for mixed shock to improve outcomes.

Patients with circulatory shock are divided into three primary hemodynamic phenotypes, namely hypovolemic, vasodilatory/distributive, and cardiogenic (including obstructive).^1^ Cardiogenic shock (CS) is a complex clinical syndrome characterized by end-organ hypoperfusion owing to a primary cardiac insult resulting in impaired cardiac output (CO).^1^, ^2^, ^3^ Traditional pathophysiologic models of CS predict that acute reductions in CO should lead to neurohormonal activation and compensatory systemic vasoconstriction to maintain mean arterial pressure (MAP), resulting in the classic CS hemodynamic profile with elevated systemic vascular resistance (SVR).^1^, ^2^, ^3^ However, CS is a hemodynamically diverse syndrome and SVR can vary widely in patients with CS.^4^, ^5^, ^6^, ^7^, ^8^ Accordingly, it has long been recognized that a subgroup of patients with CS displays inappropriately low SVR.^2^, ^3^, ^4^^,^^9^ The hemodynamic profile of depressed CO and inappropriate systemic vasodilation has been termed *mixed shock* to indicate the combined features of both cardiogenic and vasodilatory shock.^2^^,^^3^ Mixed shock is a heterogeneous syndrome with numerous etiologic drivers and variable clinical trajectories unified by a common clinical and hemodynamic profile. The Shock Academic Research Consortium describes mixed shock as a “high-risk, understudied subpopulation of patients with CS,” functioning as a modifier for the 4 primary categories of CS.^3^ In this review, we will discuss the current definitions, epidemiology, pathophysiology, classification, evaluation, and management of mixed cardiogenic-vasodilatory shock in the context of a new proposed classification system.

## Definition and diagnosis of mixed shock

To date, there is no uniform, evidence-based definition of mixed shock. Conceptually, mixed cardiogenic-vasodilatory shock may be pragmatically defined as hypotension with end-organ hypoperfusion (ie, overt shock) occurring in the context of both *acute cardiac insufficiency* and *systemic vasodilation*. The typical hemodynamic pattern of mixed shock includes the triad of low CO, low SVR, and normal or elevated ventricular filling pressures (Table 1); however, clinically relevant thresholds for these 3 diagnostic criteria remain ill-defined.^2^^,^^3^ In primary CS, hypoperfusion generally correlates with a cardiac index < 2.2 L/min/m^2^, which is a commonly used diagnostic threshold in the absence of vasopressors, inotropes, or temporary mechanical circulatory support (MCS).^2^, ^3^, ^4^ The pulmonary artery catheter (PAC) is the gold standard for diagnosis of mixed shock, allowing direct measurement and calculation of all relevant hemodynamic parameters and defining mixed shock is difficult without invasive hemodynamic monitoring. Once the CO is measured or estimated, SVR can be calculated based on the MAP—a low SVR has been defined as <700 dyn/cm/s^-5^ (normal 800-1,200 dyn/cm/s^-5^), but must be interpreted in the context of vasoactive drug support (ie, a normal SVR during vasopressor therapy may reflect inappropriate vasodilation).^2^, ^3^, ^4^, ^5^^,^^8^ Because a high SVR is typical of CS, it could be argued that mixed shock is present in any patient with CS who continues to require high vasopressor doses to maintain a normal MAP and SVR after restoration of a normal CO using inotropes and/or temporary MCS.^8^

**Table 1:** Potential Diagnostic Criteria for Mixed Shock

Diagnostic Modality	Low Cardiac Output	Systemic Vasodilation	Adequate Preload/Congestion
Pulmonary artery catheter	Low cardiac output Low mixed SVO2	Low systemic vascular resistance	Normal/elevated right atrial pressure/pulmonary capillary wedge pressure
Central venous catheter	Low central SVO2		Normal/elevated central venous pressure
Pulse contour monitoring	Low cardiac output	Low systemic vascular resistance	Low pulse pressure variation/stroke volume variation
Doppler echocardiography	Low cardiac output based on LVOT velocity-time integral	Low systemic vascular resistance	Dilated inferior vena cava Elevated mitral E/e’ ratio
Physical examination^a^	Cool extremities Narrow pulse pressure Weak distal pulses	Warm extremities Wide pulse pressure/low diastolic blood pressure Preserved distal pulses	Pulmonary congestion/edema Elevated jugular venous pressure/jugular venous distension
Support	Need for inotropes/MCS	Need for high-dose vasopressors	Need for diuretics

LVOT = left ventricular outflow tract; MCS = mechanical circulatory support; SVO_2_ = venous oxygen saturation.

The 3 clinical features that define the mixed shock phenotype are low cardiac output, vasodilation, and adequate preload/congestion. These can each be identified using various evaluation modalities.

a Physical examination findings in mixed shock may be contradictory and must be interpreted in the context of the primary shock phenotype.

## Proposed subgroups and common phenotypes of mixed shock

**Figure 1:**
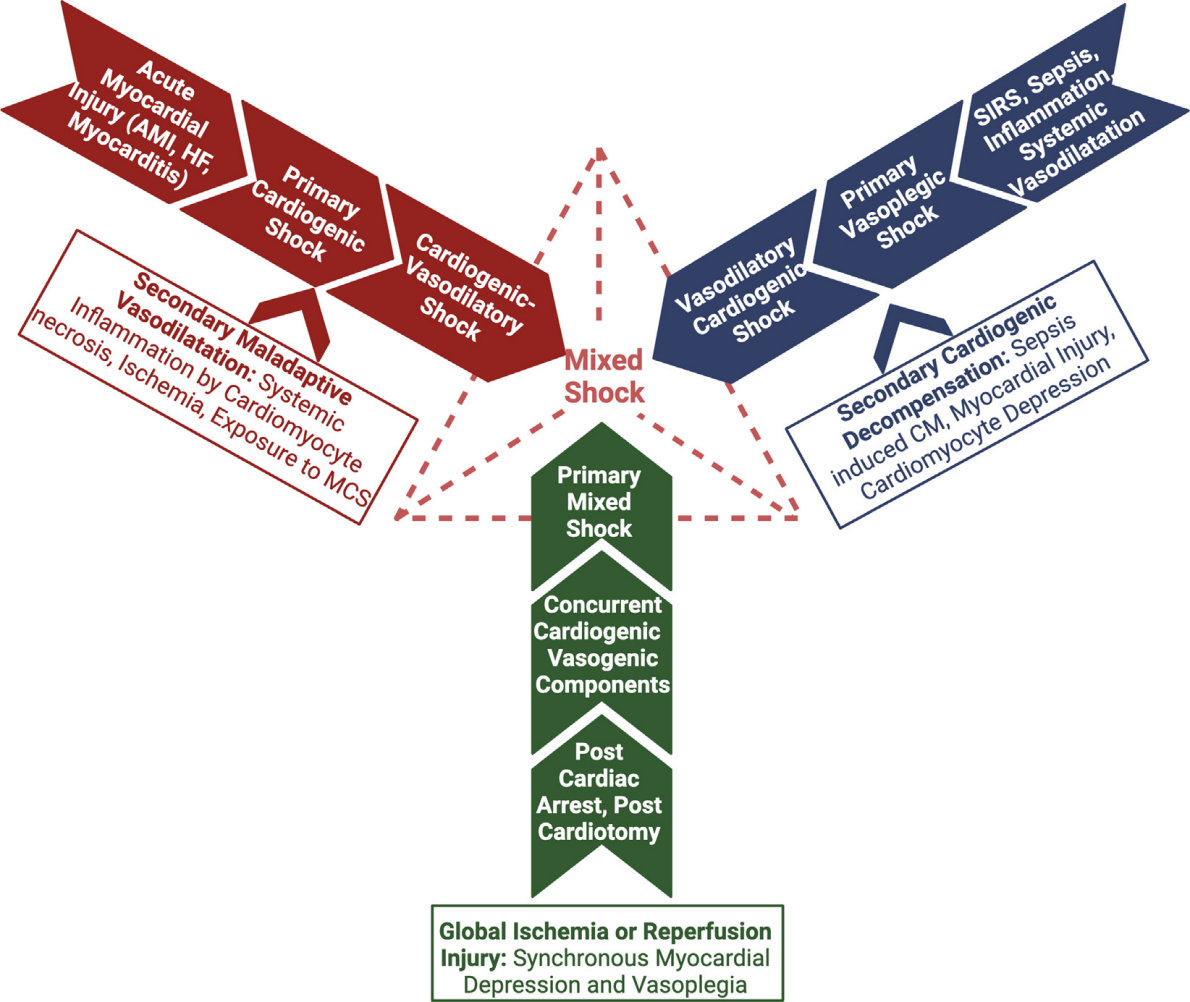
**Proposed Classification of Mixed Shock** Patients are classified based on the primary (inciting) shock phenotype and the secondary (complicating) process in a “two-hit” model. This model is conceptual, recognizing that there may be overlap between groups and it may not be possible to clinically classify patients who present late during mixed shock. HF = heart failure; MCS = mechanical circulatory support; SIRS = systemic inflammatory response syndrome.

Patients can arrive at a mixed shock state through several pathways, often starting with a primary insult causing shock followed by activation of secondary pathophysiological mechanisms or development of an intercurrent disease process. By analogy to the taxonomy of cardiorenal syndromes, we herein delineate a conceptual model that categorizes mixed shock into 3 principal groups, each defined by the sequence and nature of the insult (ie, primary versus secondary hemodynamic process): cardiogenic-vasodilatory, vasodilatory-cardiogenic, and primary mixed shock (Figure 1). The cardiogenic-vasodilatory shock group includes those patients who initially have CS (primary pathology) and subsequently develop pathologic vasodilation (secondary insult), classically patients with acute myocardial infarction (AMI) CS who develop inflammatory vasodilation. The vasodilatory-CS group includes those patients who initially have vasodilatory shock (primary pathology) and subsequently develop cardiac compromise with low output (secondary insult), for example, patients with septic shock who develop severe sepsis-induced cardiomyopathy (SICM). The primary mixed shock group includes those patients who have a single primary insult that compromises both cardiac (ie, low CO) and vascular (ie, vasoplegia) function, such as patients with postcardiac arrest syndrome (PCAS) causing myocardial dysfunction and vasodilation. Patients in the cardiogenic-vasodilatory and vasodilatory-CS categories have 2 distinct pathophysiologic mechanisms or disease processes (ie, a “two-hit” model), whereas those with primary mixed shock have a single insult that produces both myocardial dysfunction and vasodilation, either simultaneously or sequentially (Central Illustration). While we describe the distinctions between these proposed subgroups as concrete for the purposes of illustration, they may overlap substantially and be difficult to cleanly distinguish in clinical practice and research.

**Central Illustration:**
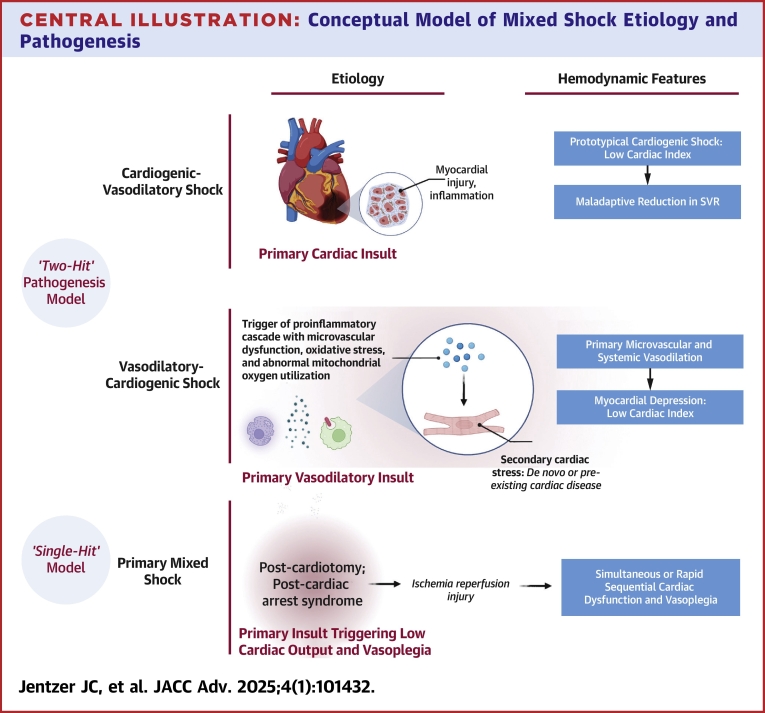
Conceptual Model of Mixed Shock Etiology and Pathogenesis Abbreviation as in Figure 2.

## Cardiogenic-vasodilatory shock

### Definition

Patients with cardiogenic-vasodilatory shock have a primary cardiac disease process resulting in low CO and prototypical CS, and then subsequently develop inappropriately low vascular tone resulting in mixed shock. The defining criterion of this group is the initial development of pure CS with later development of vasodilation and mixed shock, recognizing that patients may present late to medical care with established mixed shock. Inappropriate vasodilation causing cardiogenic-vasodilatory shock counteracts the compensatory neurohormonal response and therapeutic response to vasopressors resulting in a maladaptive reduction in SVR and progressive hypotension in CS.^2^^,^^4^^,^^10^ The development of inappropriate vasodilation often represents an inflection point in the clinical course of CS, with delayed clinical worsening and initiation of the downward spiral which will progress to refractory shock with poor outcome if not addressed promptly.^2^^,^^4^ The greater the severity of CS, the more likely that mixed shock is present (particularly in refractory Society for Cardiovascular Angiography and Interventions stage E CS).^11^, ^12^, ^13^, ^14^, ^15^

### Potential etiologies

Any condition causing CS can potentially evolve into mixed cardiogenic-vasodilatory shock from intercurrent infection or through noninfectious inflammatory disease mechanisms potentially aggravated by vasodilatory effects of medications.^3^ AMI serves as a prototypical example whereby acute myocardial injury causes pump failure and triggers a systemic inflammatory response provoking vasoplegia.^4^^,^^16^ Mirroring the overall epidemiology of CS, AMI and decompensated heart failure are the usual causes of mixed cardiogenic-vasodilatory shock.^17^^,^^18^ Acute myocarditis may present with an initial low-output state, but if excessive systemic inflammation occurs then cardiogenic-vasodilatory shock can occur, providing a clear link between inflammation and simultaneous cardiac and vascular dysfunction which may overlap with primary mixed shock.

### Epidemiology of mixed cardiogenic-vasodilatory shock

A seminal report from the SHOCK (Should We Emergently Revascularize Occluded Coronaries for Cardiogenic Shock) trial demonstrated that approximately 1 in 6 patients with AMI-CS had a low SVR (ie, <700 dyn/cm/s^−5^) reflecting mixed shock with systemic vasodilation despite use of vasopressor therapy.^5^ Baldetti et al found that 1 in 4 patients who initially had “pure” CS subsequently developed mixed shock (vasodilation and systemic inflammation) after a median of 5 days, and these patients were sicker with higher mortality; predictors of developing mixed shock included lower systolic pressure, liver injury, and infection.^19^ In an analysis from the Critical Care Cardiology Trials Network registry, physician-assigned mixed shock accounted for 20% of all shock cases treated in tertiary care cardiac intensive care units (CICUs).^17^ Compared to patients with pure CS, mixed CS patients had worse indices of acute illness severity, including more systemic hypoperfusion and end-organ dysfunction, resulting in more intensive care unit resource utilization, longer CICU stays, and higher in-hospital mortality.^17^ In a separate Critical Care Cardiology Trials Network analysis evaluating the prognostic significance of hemodynamic parameters in CS, low MAP and SVR were strongly associated with worse in-hospital mortality (whereas cardiac index itself was not), underscoring the adverse prognostic implications of the mixed CS phenotype.^7^ In a large single-center cohort of patients with CS by Chavez et al^8^, a low SVR (<800 dyn/cm/s^−5^) was present in 19% and incrementally associated with higher mortality. These authors defined “blunted pressor response” as either low SVR (<800 dyn/cm/s^−5^) while on vasopressors (present in 17%) or normal SVR (800-1,200 dyn/cm/s^−5^) while on high doses of vasopressors (>0.2 μg/kg/min norepinephrine; present in 9%); patients with CS and blunted pressor response had higher mortality.^8^

### Infection in patients with acute cardiovascular disease

The systemic inflammatory response syndrome (SIRS) is a nonspecific physiologic response to any major systemic infectious or noninfectious stressor that has been associated with higher disease severity and worse survival in acute cardiovascular disease.^9^^,^^11^^,^^16^ Criteria for SIRS are met in up to one-third of all CICU patients on admission, especially those with CS or cardiac arrest.^9^^,^^11^^,^^16^^,^^20^ Although SIRS often occurs without documented infection (“sterile SIRS”), patients with cardiac critical illness are at increased risk of infection.^21^ A neutrophilic leukocytosis is typical of patients with CS, and patients with CS often have elevated markers of systemic inflammation, making it challenging to separate infected and uninfected individuals.^11^^,^^15^^,^^18^^,^^22^, ^23^, ^24^ Among patients in the CICU, diagnoses of sepsis are increasingly common, predisposing to development of mixed shock.^25^^,^^26^ Sepsis is a common complication or comorbid condition for patients with CS, reported at 15% to 20% or more in clinical trials and observational studies, being associated with higher mortality.^9^^,^^17^^,^^18^^,^^27^^,^^28^ An analysis from the Mayo Clinic reported concomitant admission diagnoses of both CS and sepsis in 15% of CICU patients with shock, and these patients had higher illness severity, greater severity of shock, and worse survival.^18^

### Inflammation and vasodilation in cardiogenic shock

**Figure 2:**
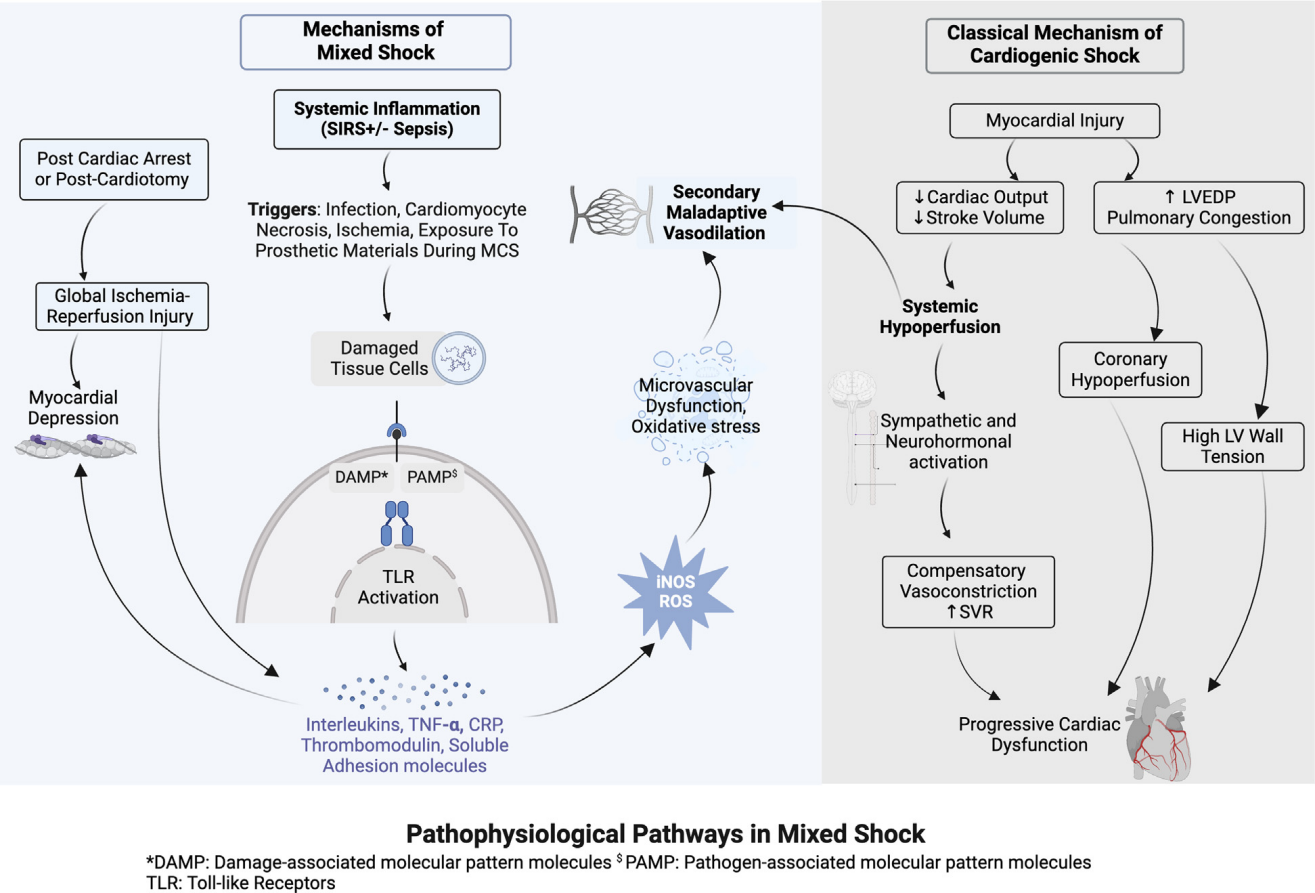
**Pathophysiological Pathways in Mixed Cardiogenic-Vasodilatory Shock, Including Classical Mechanisms of Typical Cardiogenic Shock and Established Mechanisms of Mixed Shock** Mixed shock commonly arises when tissue injury triggers a systemic inflammatory response, resulting in maladaptive vasodilation, microcirculatory dysfunction, and worsening myocardial dysfunction. ABG = arterial blood gas; CRP = C-reactive protein; LFT = liver function test; LV = left ventricular; LVEDP = left ventricular end-diastolic pressure; ROS = reactive oxygen species; SVR = systemic vascular resistance; TNF = tumor necrosis factor; other abbreviations as in Figure 1.

CS occurs when contractile failure of the heart results in reduced CO with subsequent systemic hypoperfusion and hypotension (Figure 2).^2^^,^^4^ This triggers sympathetic and neurohormonal activation with resultant compensatory tachycardia and peripheral vasoconstriction to restore CO and MAP, initially preserving organ perfusion at the expense of increased myocardial afterload and oxygen demand. Congestion aggravates myocardial dysfunction (by increasing wall tension and provoking valvular regurgitation) and organ hypoperfusion (by reducing the perfusion pressure gradient within the organs).^2^^,^^4^^,^^29^ Maladaptive vasodilation drives the transition from pure CS to mixed cardiogenic-vasodilatory shock by interfering with the compensatory vasoconstriction necessary to maintain adequate organ perfusion.^2^^,^^4^, ^5^, ^6^, ^7^

Systemic inflammation is a key pathophysiologic mechanism that drives pathogenic vasoplegia, refractory shock, and adverse outcomes in CS (Figure 2).^2^^,^^4^ Indeed, Baldetti et al defined development of mixed shock in patients who initially had “pure” CS as the development of vasodilation (“distributive hemodynamics”) in combination with evidence of systemic inflammation.^19^ The inflammatory response is initiated via activation of leukocyte toll-like receptors by binding damage-associated molecular patterns, which are derived from damaged host cells, and pathogen-associated molecular pattern, which are derived from microbial cells (eg, endotoxins).^10^ Potential triggers include infection, cardiomyocyte necrosis from AMI, PCAS, tissue ischemia, allogenic blood product transfusion, and gut translocation of bacterial endotoxins due to loss of mucosal integrity or mesenteric ischemia; exposure of the blood to prosthetic materials during MCS, particularly venoarterial (VA) extracorporeal membrane oxygenator (ECMO), is another important trigger.^2^^,^^10^^,^^30^, ^31^, ^32^, ^33^ Inflammatory cytokines and downstream pro-inflammatory targets such as various interleukins, tumor necrosis factor-α, C-reactive protein, procalcitonin, thrombomodulin, vascular adhesion molecules, circulating neutrophils, and complement are elevated in CS (particularly AMI-CS) and confer an increased risk of mortality.^10^^,^^11^^,^^15^^,^^18^^,^^22^, ^23^, ^24^^,^^34^, ^35^, ^36^, ^37^

Regardless of the trigger, the systemic inflammatory response can produce uncontrolled vasodilation through cytokine-driven excessive inducible nitric oxide synthase (NOS) activation resulting in toxic levels of nitric oxide (NO), which produces vasoplegia, microvascular dysfunction, oxidative stress, and abnormal mitochondrial oxygen utilization.^2^^,^^4^^,^^10^^,^^32^^,^^38^, ^39^, ^40^ Endothelial dysfunction and microvascular dysfunction may aggravate tissue hypoperfusion and result in capillary leak and compromised tissue perfusion despite restoration of acceptable macrohemodynamic parameters.^10^^,^^39^ Abnormalities of the microcirculation are increasingly recognized as important contributors to the pathogenesis of both CS and septic shock and thus presumably contribute strongly to vasoplegia and organ injury in mixed shock.^41^ An inadequate vascular response to catecholamines (either endogenous or exogenous) is a central mechanism contributing to vasoplegia via acidemia, reduction of intracellular calcium, elevated intracellular cyclic guanosine monophosphate/cyclic adenosine monophosphate levels and activation of adenosine triphosphate-sensitive potassium channels.^40^ As in critically ill patients with sepsis, nearly one-third of patients with CS may develop critical illness-related corticosteroid insufficiency; relative deficiencies of vasopressin and angiotensin-II have been proposed in septic shock and might potentially contribute to refractory vasoplegia in mixed cardiogenic-vasodilatory shock.^40^^,^^42^^,^^43^ Sedative and analgesic medications commonly used for mechanically ventilated patients have vasodilatory and cardioinhibitory properties that can induce or aggravate a mixed shock state, as can guideline-directed medical therapies for heart failure.^3^ Finally, metabolic derangements from sustained organ hypoperfusion can compromise myocardial and vascular function, impairing the response to vasoactive medications; this has been termed hemo-metabolic shock.^12^^,^^14^^,^^44^^,^^45^

## Vasodilatory-cardiogenic shock

### Definition

Mixed vasodilatory-CS develops when a patient with a primary vasodilatory/distributive shock phenotype develops cardiac compromise. Sepsis stands as the quintessential cause of vasodilatory shock and may have infectious origins or noninfectious mimics.^46^ Numerous other less common causes of vasodilatory/distributive shock that can progress to mixed vasodilatory-CS exist, including anaphylaxis and adverse drug reactions. Patients with sepsis as the primary pathology can develop low CO as a secondary insult through a multitude of pathways, resulting in vasodilatory-CS.^47^^,^^48^ Initially, patients present with systemic vasodilation and subsequently develop signs of cardiac dysfunction indicative of low CO due to inability of the cardiovascular system to compensate for the increased demand associated with the septic state or development of an acute cardiomyopathic process.

### Potential etiologies

Patients with pre-existing heart disease, such as cardiomyopathy or severe valvular heart disease, may be relatively compensated at baseline but deteriorate after fluid loading and systemic stress, becoming unable to maintain a normal CO during the sepsis state. New cardiac dysfunction can occur via SICM, demand ischemia (ie, type 2 AMI), stress cardiomyopathy (apical ballooning/takotsubo syndrome), or development of new valve lesions (ie, infective endocarditis).^47^^,^^49^ Patients with hyperdynamic sepsis or stress-induced cardiomyopathy can develop inducible left ventricular outflow tract (LVOT) or mid-cavity obstruction resulting in low CO despite preserved left ventricular ejection fraction.^47^^,^^49^ For most patients, SICM does not lead to mixed vasodilatory-CS, and objective evidence of inappropriately low CO should be present to define the mixed shock syndrome. Infective endocarditis is a unique cause of mixed vasodilatory-CS, whereby an initial septic shock can be complicated by CS due to valvular destruction. Mixed vasodilatory-CS may arise in severe systemic infections where direct viral myocardial injury, hypoxia-induced myocardial dysfunction, systemic inflammation, microvascular thrombosis, and endothelial dysfunction converge, causing both cardiogenic and vasodilatory shock components.

### Epidemiology of septic cardiomyopathy

SICM involves reversible myocardial dysfunction occurring among patients with sepsis, with a variable prevalence of up to 60%.^47^^,^^50^ There are multiple potential manifestations (phenotypes) of SICM, each of which has distinct epidemiology and prognosis. However, the prevalence of true mixed cardiogenic-vasodilatory shock in septic patients is not well-defined and most patients with SICM have preserved CO.^47^ In one recent study, 10% of patients with septic shock had a low cardiac index (defined as <1.85 L/min/m^2^), and a U-shaped relationship was observed between cardiac index and short-term mortality.^51^ Classic SICM includes transient left ventricular (LV) dilation and/or systolic dysfunction, which is common (up to 60%) and might represent an adaptive response to maintain CO during stress.^47^ Development of hyperdynamic systolic function with LVOT or mid-cavitary obstruction occurs infrequently and could potentially compromise CO.^47^ Abnormalities of RV structure and function occur in up to 30% to 60% of patients with sepsis, typically in the context of *cor pulmonale* from severe respiratory failure, and may occasionally impair CO.^47^

### Cardiac dysfunction in vasodilatory shock

Several pro-inflammatory cytokines along with NO and reactive oxygen species have direct myocardial depressant effects through reductions in intracellular calcium, in addition to direct myocyte injury from inflammatory mediators and immune cells; these are core mechanisms driving myocardial dysfunction in SICM (Figure 2).^10^^,^^47^ Elevated dipeptidyl dipeptidase-3 and adrenomedullin are associated with impaired cardiac contractility and worse outcomes in patients with CS and sepsis; these biomarkers could contribute to mixed shock and have been proposed as targets for therapy.^52^, ^53^, ^54^, ^55^ Stress-induced cardiomyopathy (eg, apical ballooning or takotsubo syndrome) can occur during the course of any acute illness, resulting in ventricular systolic dysfunction and a risk of LVOT obstruction.^49^ A sympathetic surge resulting in excessive activation of cardiac beta-adrenergic receptors causing direct myocardial toxicity and downregulation of contractile function is a widely accepted theory for classic stress-induced cardiomyopathy; additional mechanisms include inflammation and microvascular dysfunction or spasm.^49^ While most patients with stress-induced cardiomyopathy have preserved CO, a low-output state can occur leading to mixed shock when triggered by sepsis. Finally, demand myocardial ischemia and infarction can be triggered by vasodilatory shock and if severe can compromise cardiac function resulting in mixed shock.

## Primary mixed shock

### Definition

Primary mixed shock is characterized by development of both cardiogenic and vasodilatory shock components triggered by a single disease process. Several primary systemic insults can result in either simultaneous or sequential development of both low CO and vasoplegia, resulting in primary mixed shock. For some of these conditions, the hemodynamic shock phenotype evolves from purely cardiogenic to purely vasoplegic, with mixed shock being a temporary intermediate state.

### Potential etiologies

The PCAS arising after resuscitation from cardiac arrest and postcardiotomy syndrome are by far the most common causes of primary mixed shock.^56^, ^57^, ^58^ These conditions are both characterized by myocardial systolic dysfunction which occurs early in the clinical course and is followed by vasoplegia, often with a component of myocardial diastolic dysfunction and relative hypovolemia due to capillary leak. These typically present initially with a low-output state that may respond transiently to volume administration but then requires inotropic support and progresses to vasoplegia which may persist even after myocardial dysfunction has improved. Overdose of several individual drugs (eg, non-dihydropyridine calcium channel blockers, vasodilating beta-blockers, alpha-2 agonists, and tricyclic antidepressants) or a combination of different drugs can trigger simultaneous CS and vasoplegia.^59^ Fulminant acute myocarditis, infective endocarditis, or severe systemic infections can produce simultaneous cardiogenic and vasodilatory effects, manifesting as primary mixed shock.

### Postcardiac arrest syndrome

Cardiac arrest is common in patients with CS, either as a cause, consequence, or complicating adverse effect modifier resulting in more severe shock, greater organ failure severity, a higher prevalence of SIRS, and worse outcomes.^18^^,^^20^^,^^58^ Cardiac arrest is an important trigger for primary mixed shock with its own unique mechanisms. The stereotyped PCAS occurring after resuscitation from cardiac arrest includes both cardiac and vascular dysfunction that can trigger de novo mixed shock or produce a mixed shock state in patients who initially had cardiogenic or vasodilatory shock.^30^^,^^56^ PCAS includes postarrest myocardial dysfunction (PAMD) in most patients, and the combination of PAMD and SIRS can result in an evolving shock phenotype including mixed shock.^30^^,^^56^ PAMD is characterized by acute reversible myocardial stunning with both systolic and diastolic dysfunction superimposed on any pre-existing cardiac pathology.^56^ Global systemic ischemia-reperfusion injury (IRI) is the main pathophysiologic mechanism that drives the PCAS, affecting the heart, brain, and other organs.^58^ During the low-flow state of cardiac arrest, systemic hypoxia produces accumulation of intracellular sodium and calcium, and after reperfusion this can be aggravated by excessive production of reactive oxygen species; collectively, this triggers harmful intracellular mechanisms including programmed cell death pathways.^30^ Additional contributors to reversible PAMD include defibrillator shocks, the toxic myocardial effects of high-dose epinephrine, negative inotropic effects of anti-arrhythmic drugs, myocardial ischemia, and adverse myocardial effects of therapeutic hypothermia.^56^ A delayed SIRS response is typical of the PCAS and is triggered by IRI and other factors (often including infection), further worsening PAMD and producing vasoplegia.^30^ Elevated inflammatory markers are common in patients with PCAS, and generally correlate with the severity of shock and need for vasopressors, predicting worse outcomes.^33^^,^^60^, ^61^, ^62^, ^63^ PAMD tends to be an early phenomenon (during the first 6-24 hours), while vasodilation from SIRS tends to occur later (after 12-24 hours) as patients progress from pure CS to mixed shock to pure vasodilatory shock.^56^

### Postcardiotomy shock

Postcardiotomy shock, occurring in 5% to 20% of patients after cardiac surgery (depending on the definition) and triggered by cardioplegia and prolonged cardiopulmonary bypass, carries substantial clinical and pathophysiologic similarities to the PCAS.^3^^,^^57^ In both circumstances, myocardial and systemic IRI occurs and often triggers a SIRS response with concomitant cardiac dysfunction and vasoplegia.^56^ Postcardiotomy shock may include unique features distinct from PAMD, such as predominant right ventricular dysfunction (eg, from coronary air embolism) and the effects of opening the pericardium on cardiac function.^57^

## Diagnostic evaluation of mixed shock

### Clinical features of mixed shock

Patients with mixed shock typically present with severe or worsening shock including vasopressor-dependent hypotension and clinical and laboratory evidence of hypoperfusion and end-organ dysfunction. Usually, a patient will start with typical signs of either cardiogenic or vasodilatory shock and then develop evidence of poor or worsening perfusion and escalating need for hemodynamic support. A key indicator of the evolution to mixed shock is reversal of expected clinical and hemodynamic findings compared to the initial diagnosis of either cardiogenic or vasodilatory shock, or the presence of conflicting findings regarding the assessment of CO and SVR. The central or mixed venous oxygen saturation (SVO_2_) can be a useful biomarker indicating the development of mixed shock but must be interpreted in the context of arterial oxygenation, hemoglobin, and metabolic demands. For patients with an initial cardiogenic phenotype, mixed shock may be heralded by the development of an unexpectedly high or rising SVO_2_ or physical examination findings suggesting vasodilation (eg, warm extremities, strong distal pulses, and wide pulse pressure with low diastolic pressure). Conversely, for patients with an initial vasodilatory phenotype, mixed shock may be heralded by the development of an unexpectedly low or dropping SVO_2_ or physical examination findings suggesting vasoconstriction (eg, cool extremities, weak distal pulses, and narrow pulse pressure with low systolic pressure). Primary mixed shock often evolves from one phenotype to the other, with changing clinical findings over time.

Most patients with mixed shock have evidence of systemic inflammation, with neutrophilic leukocytosis, a high neutrophil-to-lymphocyte ratio, and elevated levels of inflammatory markers (eg, C-reactive protein and cytokines); fevers may be present, but none of these findings is specific for infection.^9^^,^^11^^,^^15^^,^^17^, ^18^, ^19^^,^^34^ Patients with mixed shock should be evaluated thoroughly for infection, with blood and (if indicated) urine or respiratory cultures, appropriate imaging, and prompt initiation of empiric broad-spectrum antimicrobial therapy until infection can be ruled out; potential sources of catheter and line-related infection should be removed if possible.^21^^,^^46^

### Value of hemodynamic monitoring in mixed shock

The diagnosis of mixed shock can be difficult to make without invasive hemodynamic monitoring to measure CO and SVR directly. A PAC is indispensable for providing minute-to-minute real-time invasive hemodynamic monitoring including biventricular filling pressures, CO, and SVR to assess the response to treatment and adjust therapies to achieve therapeutic goals. As in all patients with severe or worsening CS, PAC placement is advisable and represents the gold standard for diagnosis in mixed shock.^2^ Use of PACs, particularly when complete hemodynamic data are obtained, has been associated with better outcomes in CS patients in observational studies.^6^^,^^64^ When a PAC is not available, CO may be estimated using minimally invasive (arterial pulse contour monitoring) or noninvasive (echocardiography) testing and the SVR can be extrapolated based on these estimated CO values.

### Cardiac assessment in mixed shock

For patients with evidence of mixed shock (particularly vasodilatory-CS), an evaluation for new or worsening cardiac dysfunction is indicated. Evaluation for myocardial ischemia using cardiac biomarkers and electrocardiography should be pursued. Bedside echocardiography is a crucial diagnostic test for identifying wall motion abnormalities which may indicate ischemia, in addition to providing invaluable information regarding cardiac structure and function to identify potential contributors to impaired cardiac performance. Abnormal ventricular systolic function is neither necessary nor sufficient to confirm a reduced CO, and a focused hemodynamic assessment is essential for the diagnosis.^47^^,^^50^ When acute myocardial ischemia is associated with new or worsening cardiac dysfunction, coronary angiography should be considered.^2^

## Management of mixed shock

### Fluid management

For patients with mixed shock, an assessment of preload-responsiveness can be followed by a cautious crystalloid bolus (eg, 3-4 ml/kg) in the absence of manifest congestion, recognizing that static filling pressures alone are a poor marker of volume response.^2^^,^^65^ Many patients with vasodilatory-CS have already received volume resuscitation for sepsis (ie, guideline-congruent 30 ml/kg), and in some cases diuresis may be needed to treat congestion.^46^ After initial resuscitation, conservative or restrictive fluid management is beneficial in most critically ill patients, and likely to be appropriate in patients with mixed shock.^66^

### Vasoactive medications

**Figure 3:**
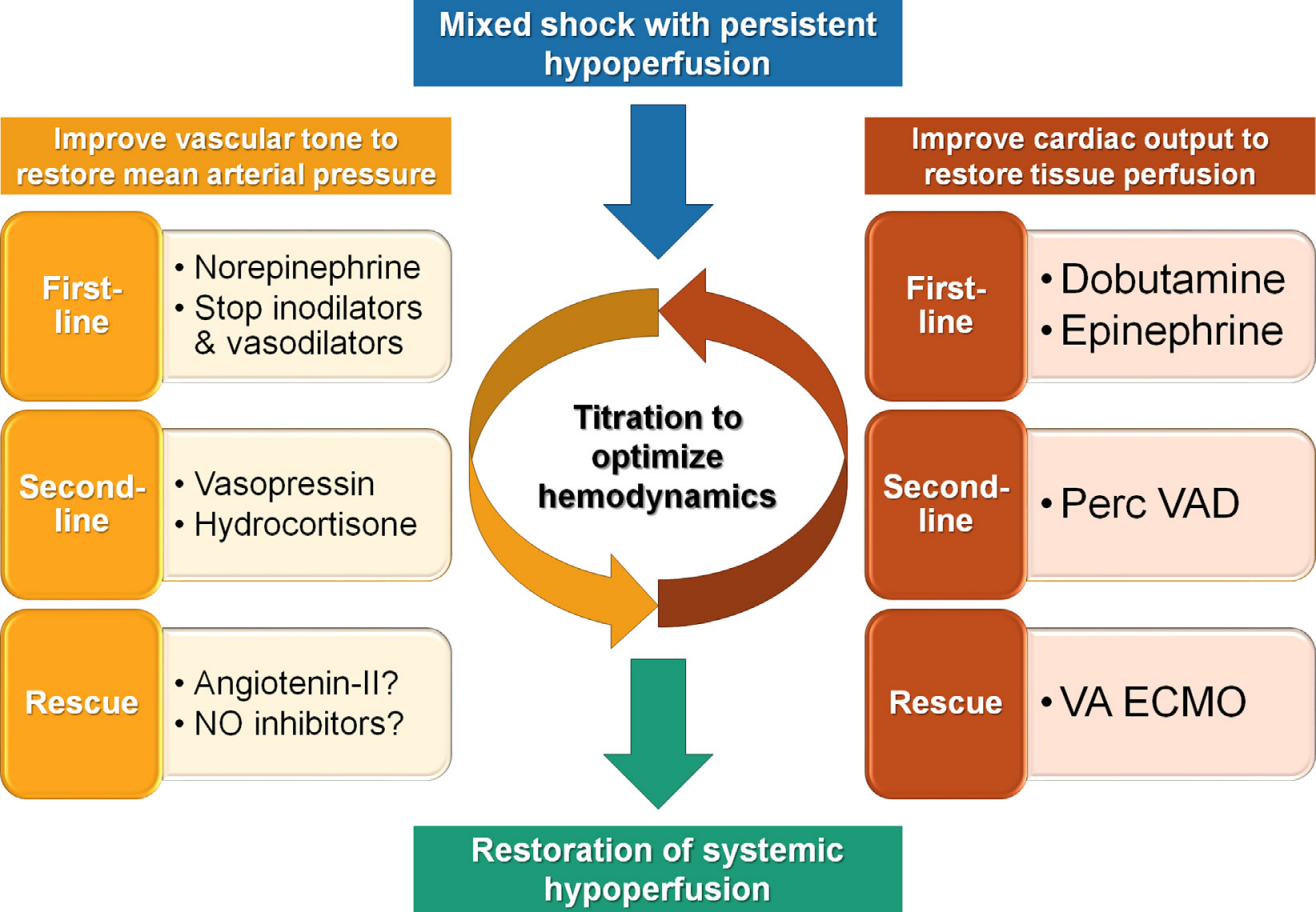
**Overview of Hemodynamic Support in Mixed Shock** Vasopressors (drugs that cause vasoconstriction and increase systemic vascular resistance) and adjunct drugs that inhibit vasodilation are titrated to restore mean arterial pressure, while inotropes (drugs that stimulate myocardial contractility) and mechanical circulatory support are titrated to restore cardiac output VA. ECMO = venoarterial extracorporeal membrane oxygenator.

There are no randomized trials examining patient-centered outcomes for patients with mixed shock, forcing us to extrapolate from patients with cardiogenic or septic shock in whom vasoactive medications are ubiquitously used to restore adequate systemic hemodynamics and perfusion.^1^^,^^2^^,^^67^ Titrating vasoactive medications to restore hemodynamics is facilitated by the use of invasive hemodynamic monitoring to guide therapy in patients with mixed shock. Separate titration of inotropes (drugs that increase myocardial contractility) to adequate CO and vasopressor agents (drugs that increase SVR via vasoconstriction) to adequate MAP is suggested (Figure 3).^67^ Discontinuing or reducing the dose of medications that can aggravate the mixed shock state is essential, including inodilator or sedative drugs.^67^

**Figure 4:**
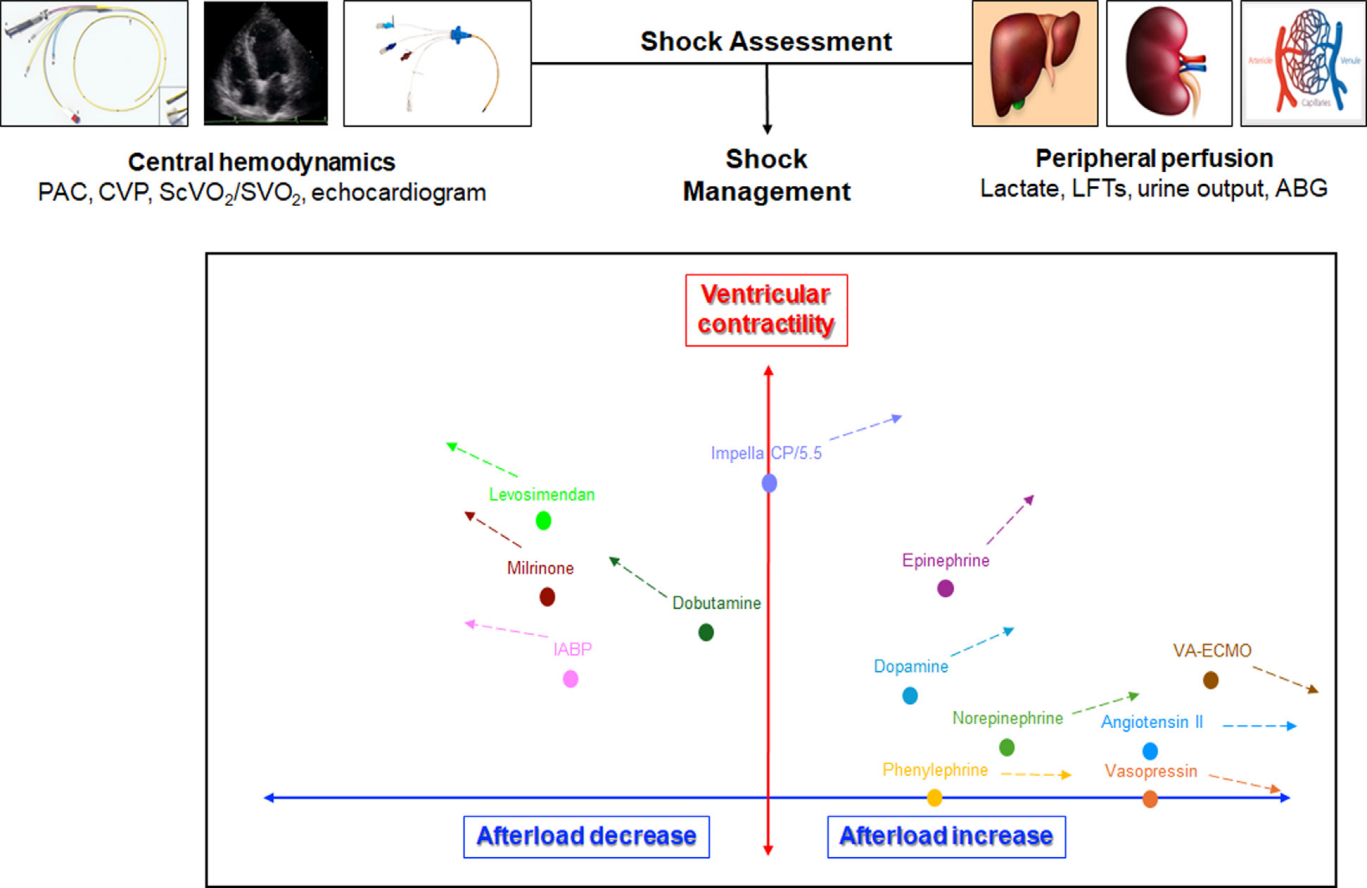
**Integration of Central Hemodynamics and Perfusion in Selection of Support Therapies in Mixed Shock** The vascular effects of vasoactive drugs must be considered when selecting inotropes and vasopressors in patients with mixed shock, as these drugs can potentially improve or worsen vasodilation or low cardiac output. PAC = pulmonary artery catheter; SVO_2_ = venous oxygen saturation; other abbreviation as in Figure 3.

The vascular (ie, vasoconstrictor or vasodilator) properties must be considered when selecting inotropic drugs in mixed shock patients whose low vascular tone makes them more susceptible to vasodilatory effects (Figure 4).^67^ Inodilators used to increase CO include dobutamine, milrinone, and levosimendan (not available in the United States).^2^^,^^67^^,^^68^ All of these drugs can trigger or exacerbate vasodilatory hypotension in patients with mixed shock, and none has demonstrated a clear advantage for patient-centered outcomes.^68^ Low dose inoconstrictors (eg, low-dose epinephrine up to 0.1 mcg/kg/min) may be added or substituted for inotropic support when an inodilator aggravates vasodilatory hypotension or for patients with vasodilatory-CS from sepsis.^46^^,^^67^ Ideally, markers of tissue perfusion such as capillary refill time should be normalized in concert with restoration of CO.^39^^,^^41^^,^^69^

Societal guidelines recommend norepinephrine as the first-choice vasopressor for both cardiogenic and septic shock, making norepinephrine an appropriate first-line vasopressor for most patients with mixed shock.^2^^,^^46^^,^^67^ Norepinephrine largely acts as a peripheral vasoconstrictor with modest inotropic properties.^67^ Randomized trials in patients with CS and septic shock have demonstrated that norepinephrine is associated with a lower risk of adverse events (eg, arrhythmias) when used as the first-line vasopressor compared with high doses of epinephrine or dopamine.^2^^,^^46^^,^^67^^,^^68^^,^^70^ The potential advantages of norepinephrine appear greater when higher vasopressor doses are required, as is often the case in mixed shock.^71^^,^^72^

Pure vasoconstrictors can be used as adjunctive catecholamine-sparing vasopressors for patients with significant vasoplegia, including vasopressin, angiotensin-II, and phenylephrine.^40^^,^^46^^,^^67^ The main limitation of these drugs is the reduction in CO that can occur via increased afterload and sympathetic withdrawal, and they should only be used after CO is normalized.^67^ Phenylephrine and vasopressin are particularly useful in vasodilated patients with hyperdynamic LV function and LVOT obstruction.^73^ For other patients with mixed shock and severe vasoplegia, addition of a pure vasoconstrictor can be considered when high-dose norepinephrine (eg, >0.2 μg/kg/min) is ineffective or associated with arrhythmias.^40^ In general, vasopressin is the preferred second-line vasopressor for most vasoplegic states including sepsis and the postcardiotomy syndrome.^40^^,^^46^^,^^57^^,^^67^^,^^74^ Limited studies suggest that vasopressin may be able to improve MAP in patients with severe CS, implying a role in mixed cardiogenic-vasodilatory shock.^75^^,^^76^ We rarely use phenylephrine, which tends to cause excessive vasoconstriction and tissue ischemia and has less hemodynamic efficacy.^67^ Angiotensin-II can increase MAP in vasodilatory shock with high norepinephrine requirements but is contraindicated in patients with a low-output syndrome.^40^^,^^67^^,^^77^ Addition of angiotensin-II to norepinephrine and vasopressin may have favorable effects on the kidneys in patients with severe vasoplegia but can increase RV afterload unlike vasopressin.^40^^,^^77^

When combination vasopressor therapy is inadequate to restore vascular tone and MAP, adjunctive agents are considered. While several of these therapies have been shown to improve MAP in patients with cardiogenic or septic shock, none has been shown to improve survival and they are understudied in mixed shock leaving uncertainty about when they should be utilized. The most commonly described adjunct for vasoplegia is stress-dose hydrocortisone (eg, 200 mg/day), which can improve MAP and reduce vasopressor requirements in patients with severe septic shock; however, improvement in patient-centered outcomes remains uncertain.^43^^,^^46^ Given the potential for relative adrenal insufficiency in patients with CS, it is possible that stress-dose corticosteroids might improve vasoplegia in mixed cardiogenic-vasodilatory shock.^42^ There is uncertainty about how to identify patients who are likely to respond to corticosteroids, making empiric therapy reasonable for patients with mixed shock who require high vasopressor doses or multiple vasopressors.^40^^,^^43^^,^^46^

Due to the involvement of excessive NO release as a core driver of vasoplegia, the use of NOS inhibitors has been examined in both septic and cardiogenic shock.^38^^,^^78^^,^^79^ While these agents were effective at increasing MAP and decreasing vasopressor requirements, they did not improve survival and a signal of potential harm was suggested.^38^^,^^78^^,^^79^ Nonselective NOS inhibition reduces the harmful effects of excessive systemic NO production on the macrocirculation, at the cost of blocking the potentially beneficial effects of local NO production on the microcirculation and immune function.^40^ Accordingly, systemic NO inhibition is not an accepted strategy for patients with septic or cardiogenic shock.^2^^,^^46^ Nonetheless, the use of contemporary NO inhibitors such as methylene blue and hydroxycobalamin is well described in patients with vasoplegia, particularly after cardiac surgery.^57^^,^^80^ Like their predecessors studied in septic and cardiogenic shock, these drugs can increase MAP and SVR; however, data on improving survival outcomes are lacking.^38^^,^^78^, ^79^, ^80^ Therefore, NO inhibitors are a third-line strategy when adequate vascular tone cannot be restored with multiple vasopressors at doses that do not elicit toxicity, and further data are needed to determine their safety.^40^ Correction of severe metabolic alkalosis (eg, with alkali therapy) or severe ionized hypocalcemia (eg, with calcium chloride) can transiently improve MAP in shock without clear effects on survival.^40^

### Mechanical circulatory support

The rationale for the use of MCS devices in CS is centered on their ability to improve systemic and coronary perfusion, reduce cardiac filling pressures, facilitate revascularization, reduce vasoactive medication doses, all in order to interrupt the downward shock spiral of tissue hypoperfusion, end-organ failure, and death.^2^^,^^81^ In patients with CS, MCS devices increase CO which increases MAP, although the effect on MAP may limited by vasoplegia in mixed shock. The intra-aortic balloon pump in particular functions by reducing effective LV afterload and tends to be ineffective in patients who are vasoplegic, have low SVR, and/or require high vasopressor doses (ie, those with mixed shock).^82^ MCS devices such as percutaneous ventricular assist devices or VA ECMO generate a fixed amount of flow, which may be inadequate to restore MAP for patients who are vasoplegic.

The few randomized controlled trials examining the use of temporary MCS in broad cohorts with CS have failed to demonstrate improvement in clinical outcomes, which may be due to either lack of device efficacy or limitations in trial design or implementation.^83^^,^^84^ Nonetheless, the use of temporary MCS in selected CS patients may result in improved survival.^85^ Generally, MCS is more appropriate for patients with primary CS resulting in mixed cardiogenic-vasodilatory shock, noting that mixed shock may develop or be unmasked after MCS device deployment. Device selection is best tailored to the specific etiology and hemodynamic phenotype (ideally guided by a PAC) and typically must be combined with vasoactive therapy to maintain SVR in mixed shock.^81^ A multidisciplinary “Shock Team” has been associated with better outcomes in patients with CS and could be particularly useful for patients with mixed shock of all subtypes (including those without primary CS) considering to their complex hemodynamics.^28^

Owing to its greater capacity to provide flow and support both ventricles, VA ECMO is commonly used for refractory mixed shock.^48^ Studies in patients with CS (primarily AMI-CS) have not demonstrated improved survival with early VA ECMO; patients enrolled in these studies received high doses of vasopressors and some likely had mixed cardiogenic-vasodilatory shock.^83^^,^^84^ Specific data regarding the use of VA ECMO in mixed shock comes primarily from observational studies of patients with vasodilatory-CS with severe SICM.^48^ These studies emphasize that patients with lower left ventricular ejection fraction appear more likely to benefit from VA ECMO, presumably because there is a greater cardiogenic component to their shock syndrome.^48^ Provision of adequate ECMO flows for patients with severe vasoplegia can be challenging and may require addition of a second venous drainage cannula. The use of VA ECMO is associated with a substantial risk of complications and should be reserved only for patients with refractory shock in whom less-invasive therapies fail.^48^^,^^84^

## Research priorities in mixed shock

Patients with mixed shock continue to have high morbidity and mortality, and many important scientific questions must be addressed given their unmet clinical needs (Table 2). Novel scientific approaches and emergence of newer therapies represent important strategies to mitigate the lethal burden of mixed shock. The “host response” to myocardial injury and end-organ failure in CS and mixed shock may be maladaptive and deleterious, and potential targets for therapy to limit these harmful mechanisms need to be identified.^86^^,^^87^

**Table 2:** Research Priorities in Mixed Shock

Research Domain	Potential Study Questions of Interest
Classification and epidemiology	• Standardize definitions of mixed cardiogenic and distributive shock • Elucidate mixed shock outcomes in CICU, medical intensive care unit, and other intensive care units • Develop and validate risk prediction models for short- and long-term survival
Basic and translational science	• Develop a reproducible model of mixed shock to elucidate novel pathways of injury and disease in mixed shock • Examine role and contribution of impaired microcirculation and inappropriate vasodilation
Randomized clinical trials	• Perform RCT examining first or second vasopressor agent in mixed shock (ie, norepinephrine vs epinephrine) • Design RCT examining role and timing of hemodynamics to guide initial treatment selection
Clinical research	• Identify optimal physiological and hemodynamic targets • Evaluate invasive mechanical ventilation strategies • Clarify timing and role of inotropic and vasoactive and temporary MCS support • Elucidate timing and role of renal replacement therapy
Patient safety and quality improvement	• Identify disease-based CICU quality and safety metrics • Prevent and manage critical care complications • Identify potential role of shock survivorship clinics and management of long-term sequelae
Health services and outcomes research	• Examine care delivery and staffing models • Evaluate comparative effectiveness of therapeutic strategies
Machine learning and novel scientific approaches	• Develop and validate clinically relevant clusters using machine learning/artificial intelligence approaches • Employ causal inferential methods in observational registry-based data

CICU = cardiac intensive care unit; MCS = mechanical circulatory support; RCT = randomized controlled trial.

Clear diagnostic criteria are needed to enable generation of better epidemiological data to understand the true disease burden, for example, for SICM or mixed cardiogenic-vasodilatory shock. Research priorities should include a standardized definition for mixed shock for both clinical trials and contemporary practice. Consensus definitions for CS and septic shock fail to precisely define mixed shock, which limits both registry-based research endeavors and the conduct and reporting of clinical trials.^3^^,^^46^ Perhaps an expert panel approach convening a multi-stakeholder collaboration including clinicians, investigators, industry representatives, regulators, and payers may help to generate consensus definitions for safety and outcomes in mixed shock to promote clarity and consistency in future research, analogous to the Shock Academic Research Consortium statement on CS.^3^

Mechanistic studies should further elucidate the complex pathophysiology of mixed shock by highlighting which specific cytokines and secondary signaling pathways are most important for causing the hemodynamic disturbances and adverse prognosis. It is essential to determine whether different etiologies of mixed shock (eg, cardiogenic-vasodilatory versus vasodilatory-cardiogenic) have divergent biomarker patterns and/or which final common pathways are involved in disease progression. The various mechanisms underlying SICM and inflammation-associated myocardial dysfunction have not been fully elucidated, and potential targets of therapy must be identified.^47^ Machine learning approaches have been applied in pure CS and may be useful in determining clinically relevant clusters in mixed shock; underlying biomarker patterns unmasked using machine learning could provide insights into the mechanistic differences between cardiogenic-vasodilatory and vasodilatory-CS.^44^^,^^45^

A crucial requirement is for the transition from a syndromic classification of mixed shock to defining *subphenotypes* and *treatable traits* that can be used to guide therapy.^44^^,^^45^ *Subphenotypes* link patient subgroups based on clinical and biochemical markers as well as disease pathobiology and may allow for identification and characterization of those patients, while a *treatable trait* is a component of a subphenotype that identifies an individual with a better response to a specific treatment. Presumably, different subphenotypes will require divergent diagnostic and therapeutic approaches, which could involve different molecular targets. Identification of distinct subphenotypes and treatable traits in mixed shock can be potentially used for predictive enrichment in future clinical trials.

On a practical level, the optimal approach to diagnosis, monitoring, and prognostication of mixed shock (eg, integrating noninvasive modalities such as echocardiography with invasive hemodynamic monitoring) at bedside is needed and may guide selection of therapeutic targets for hemodynamic support. Identifying the optimal MAP targets in various phenotypes of mixed shock is of utmost importance (particularly as low SVR will disproportionately lower the diastolic blood pressure and MAP), as is the optimal approach to the selection and titration of vasoactive drugs and MCS. Several adjunctive therapies have been explored to a limited extent in septic shock or CS, but not in mixed shock; most of these therapies have some effect on MAP but this has not been consistently translated to an improvement in patient-centered outcomes including mortality, necessitating randomized trials.^38^^,^^46^^,^^48^^,^^62^^,^^66^^,^^74^^,^^75^^,^^78^, ^79^, ^80^^,^^88^ The microcirculation has central importance in both cardiogenic and septic shock as a determinant of tissue perfusion and outcomes, raising questions about how best to monitor and resuscitate the microcirculation in mixed shock.^10^^,^^39^

## Conclusions

Mixed shock is a hemodynamically complex, multifactorial, heterogenous syndrome involving a diverse group of etiologies ultimately resulting in a combined hemodynamic lesion associated with greater vasopressor requirements, higher severity of illness, and worse outcomes than typical shock phenotypes. The adverse effects of systemic inflammation on cardiac and vascular function commonly drive mixed shock regardless of the cause, which may be initially cardiogenic or vasodilatory before evolving to a mixed state. Due to the lack of a consensus definition, patients with mixed shock have not been extensively studied, precluding robust analyses or evidence-based recommendations from professional societies. Invasive hemodynamic monitoring is important to identify and manage patients with mixed shock, who typically present with late decompensation in a patient with cardiogenic or vasodilatory shock. Rational combination vasopressor and inotrope therapy must be titrated judiciously based on hemodynamic monitoring, and MCS may be less effective for raising MAP in severely vasoplegic patients. Considering that patients with mixed shock often include the most critically ill patients with refractory shock, greater research efforts are needed to understand the role of adjunctive therapies in this high-risk population.

## Funding support and author disclosures

This study was sponsored by the ACC Critical Care Cardiology and Interventional Cardiology Sections.
